# Optimization of Artificial Light for Spinach Growth in Plant Factory Based on Orthogonal Test

**DOI:** 10.3390/plants9040490

**Published:** 2020-04-10

**Authors:** Tengyue Zou, Chuanhui Huang, Pengfei Wu, Long Ge, Yong Xu

**Affiliations:** College of Mechanical and Electronic Engineering, Fujian Agriculture and Forestry University, Fuzhou 350002, China; zouty@fafu.edu.cn (T.Z.); charleswonglink@hotmail.com (C.H.); WuPengFly666@163.com (P.W.); GLbaymax@163.com (L.G.)

**Keywords:** spinach, light-emitting diode, artificial light, photosynthesis, facility agriculture

## Abstract

Artificial LED source provides the possibility to regulate the lighting environment in plant factorys that use limited space to plant, aiming at high throughput and good quality. However, different parameters of light intensity, quality, and photoperiod will influence the growth and accumulation of bio-compounds in plants. In order to find the optimal setting of LED light for spinach planting, four group experiments were designed using the orthogonal testing method. According to the experimental results, for growth indexes including fresh weight, dry weight, root length and so on, photoperiod is the most influential factor, light intensity is the second, and light quality is the least. The best light mode (R:B = 4:1, photosynthetic photon flux density (PPFD) = 100 μmol∙m^−2^∙s^−1^ and 13/11 h) among all eight possible combinations in the range was also determined. Furthermore, for quality indexes, including the soluble sugar content, protein content and so on, a new scoring method was introduced to make a comprehensive score for evaluating. Then, the light combination (R:B = 4:1, PPFD = 150 μmol∙m^−2^∙s^−1^ and 9/15 h) in the range was found as the optimal scheme for spinach quality under those parameters. As there is trade-off between the optimal light parameters for growth and quality, it is necessary to achieve a balance between yield and quality of the plant during production. If farmers want to harvest spinach with larger leaf area and higher yield, they need to pay attention to the adjustment of the photoperiod and use a lower light intensity and a longer lighting time. If they do not mind the yield of the vegetable but want to improve the taste and nutrition of spinach products, they should pay more attention to the light intensity and use a higher light intensity and a shorter lighting time.

## 1. Introduction

Spinach is a major cultivated vegetable throughout the world. Due to its appreciable amount of iron content, spinach is popular in various recipes and dishes. It is usually planted in the field and illuminated by natural light. However, the intensity of the light varies with time and is not uniform throughout the day. However, with the development of agricultural engineering technology, people are constantly seeking higher yields and better quality. Therefore, intensive plant factories illuminated by artificial light sources will solve the trade-off between limited land and increasing production demand in the future. Plant factories use shelves designed to increase planting acreage, and artificial light sources are used to provide uniform, fixed-time, and specific-wavelength illumination to ensure product yield and quality.

Among various artificial light sources, light-emitting diode (LED) is the most promising in the field of plant lighting. It is proven to be able to influence the growth and reproduction of plants by adjusting the light quality, light intensity, and photoperiod [1,2,3]. It has been shown that blue and red LED illumination is effective in the growth of basil microgreens [4]. The LED lighting also affects growth, morphogenesis and phytochemical contents of *Myrtus communis L.* in vitro [5]. Recent studies found that the spectral quality of LED can even alter stomatal functioning, chilling tolerance, and pigments of some plants [6,7,8]. Therefore, the parameters of LEDs are considered to affect the growth and photosynthesis of plants, and also the yield and quality of them [9,10,11,12]. Thus, the optimal vegetable production can be achieved by adjusting the parameters of LED light quality, light intensity, and photoperiod [13,14,15]. However, as a popular vegetable, the suitable LED light parameters for spinach have not been fully investigated. Only the LED lamps with continuous spectrum and several specific light intensity levels were applied for this plant [16,17]. The quality of the spinach has not been investigated in those studies. Thus, in order to discover the effects of light on the growth and quality of spinach, experiments were designed in this paper using the orthogonal test [18]. The light intensity, quality, and photoperiod were all considered in the analysis. The orthogonal test is widely used as a powerful tool for experimental design [19,20,21]. It has many advantages, such as the reduction of the experimental treatments and easiness of analyzing the results [22]. Furthermore, to make the experiments more accurate, a new plant growth shelf with a uniform light environment was designed, and a new scoring method was introduced to calculate a comprehensive score for evaluating quality indexes of the plant. After the experiments, the optimal light environment for growth and quality indexes of spinach was recommended, separately.

## 2. Materials and Methods

### 2.1. Plant Material

The cultivar of the spinach used in our experiments is called “Disease-resistant 388”, originated from the Netherlands, and provided by Huanuo Corporation in Shouguang, Shandong province, China. The plants of spinach were cultivated from September to November in Fujian Agriculture and Forestry University, Fuzhou, China. The plant tray size is L60 * W40 * H13 cm. Each tray is planted with 48 spinaches as 6 rows and 8 columns and equipped with aeration pump to ensure oxygen supply to the roots.

### 2.2. Culture Device

**Figure 1:**
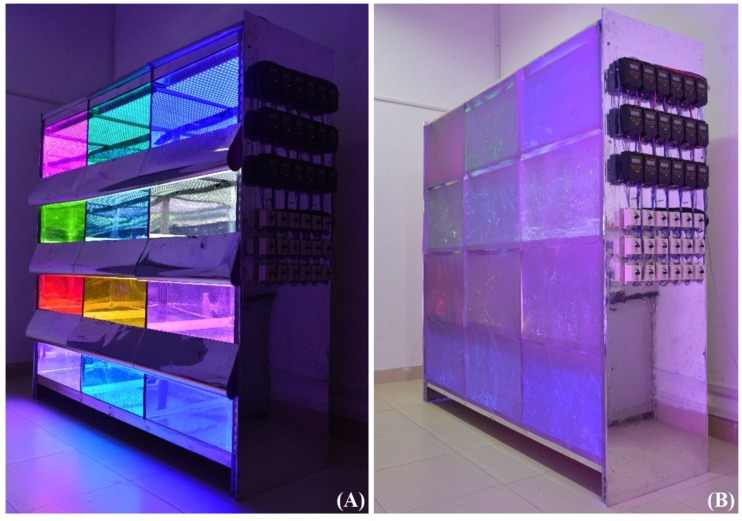
(**A**) Frontview of the experimental shelf; (**B**) frontview with the reflective curtains and control devices for the chamber.

**Figure 2:**
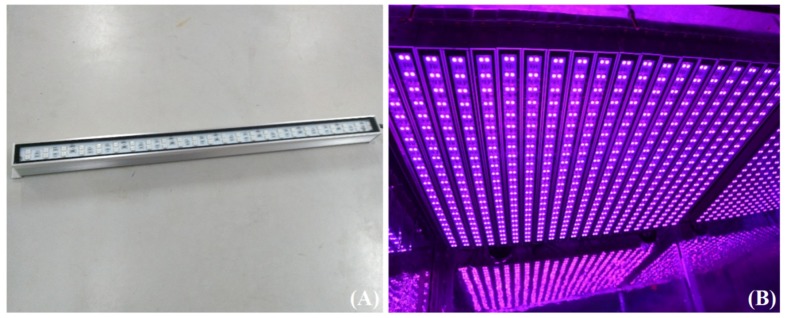
(**A**) A LED lamp used in the experiment; (**B**) the LED array on the top of each chamber.

In order to carry out the research on the light environment of plant growth, a new LED plant growth shelf was developed to optimize the light efficiency and maximize the light intensity. As shown in Figure 1, the shelf is composed of four layers with three L65 * W60 * H35 cm chambers on each layer. The distance between light and canopy areas can be calculated by subtracting the current height of the spinach plant. On the top of each chamber, 21 waterproof light-emitting diode (LED) linear lamps, made by Kedao Technology Corporation (Huizhou, China) with the type of UH-BLDT0510, are uniformly distributed, as shown in Figure 2. The size of the LED lamp is 500 * 28 mm, with voltage 24 V DC and power consumption of 11 W. A single LED lamp integrates 48 beads, each having three LED chips with different colors, including red, blue and green, which is a cool light source. The peak wavelengths of red, blue, and green light are 660, 460, and 525 nm, respectively. Furthermore, the half-wave width of each color is 20 nm. It is worth mentioning that as the spinach grew in a spreading way; there was always enough space between the canopy of the spinach and the LED lamps during the whole process of our experiments.

The Programmable Logic Controller (PLC) was used to control the intensity of each color’s LED. An air conditioner made by Gree Corporation (Zhuhai, China) and a humidifier from Lqnir Corporation (Shanghai, China) were deployed to regulate the temperature and humidity for the whole shelf. Each cultivation chamber was also equipped with two small fans for air circulation. Polished stainless steel plate, the reflectance of which to visible light is 65%, was attached on the internal walls of each chamber to reduce light loss and to make the illumination in the chamber as uniform as possible.

### 2.3. Culture and Light Treatments

The seeds of spinach were soaked in clean water for 3 h and then transferred to a thermostatic chamber made by Baihui Corporation (Guangzhou, China) at 22 °C for germination. After the germination rate reached 80%, the seedlings were moved out and sowed to the tray. The ratio of the medium for the seedlings in the plug tray was peat ash:vermiculite:perlite = 3:1:1. After the plants grew to three-leaf and one-heart, the seedlings which grew vigorously and without disease were selected and moved into the indoor environment. Then the seedlings were transplanted into the self-developed LED plant growth chambers after 2 days. The Japanese garden test nutrient solution was used for culturing [23]. The plants were planted in well-designed foam plates, and the roots were covered with sponge strips to ensure the supply of the culture solution. The temperature in the laboratory was maintained at 20 °C, the relative humidity was 80%, and the pH and EC values of the culture solution were measured every 24 h. If the pH was too low, an appropriate amount of NaHCO_3_ solution was added for adjustment. The light environment in the cultivation chamber was measured every 3 days for correction. The culture solution was changed every 7 days, and the height of the liquid surface was adjusted according to the growth conditions of the plants.

### 2.4. Data Collection

*Growth indexes* include the dry weight and fresh weight of the aerial parts, water content, root length, root surface area, and the area of the largest leaf. The fresh weight was determined immediately after removing the plants from the chamber. The dry weight was measured after the plants were oven-dried at 105 °C for 15 min and then dried at 60 °C for 48 h. The root and leaf measurements were performed through machine vision analysis using the WinRHIZO Root Analysis System from Regent Instruments Inc. (Quebec, Canada) in conjunction with an EPSON Expression 11000XL scanner (Nagano-ken, Japan).

*Quality indexes* include the soluble sugar content, protein content, malondialdehyde content, and chlorophyll content. The freshly picked appropriate leaf samples were taken, and the aging part of the leaf, which showed yellow color, was removed before cutting and mixing for the measurement of the quality indexes. The soluble sugar content was measured by the Anthrone colorimetry [24]. Protein content was measured by the Bradford method [25]. Malondialdehyde (MDA) content is an important indicator to measure the strength of plants in response to adverse conditions, which was determined by the absorbance method [26]. The actual operation is presented as follows.

The fresh spinach leaves were crushed and mixed. Then, 0.5 g of it was weighed and put into a mortar. After that, 5 mL of 5% trichloroacetic acid solution and a small amount of quartz sand were added to fully grind them, and this was repeated three times. Next, the extract was put into a centrifuge tube, and it was placed in a 3000 r/min H1850 high-speed freezing centrifuge from Cence Corporation (Changsha, China) for 10 min. Then, 2 mL of the supernatant was taken into a test tube after centrifugation. Afterwards, 2 mL 0.67% thiobarbituric acid was added, and the solution was shaken and placed in a water bath at 100 °C for 30 min. It was taken out, cooled into the room temperature, and then centrifuged again. Lastly, 3 extracts were taken after centrifugating twice, and the absorbance at 600, 532, and 450 nm was measured using an ultraviolet-visible spectrophotometer. The malondialdehyde content could be worked out by the following equation.

(1) $$\mathrm{Content}\;\mathrm{of}\;\mathrm{malondialdehyde}\;(\mu \mathrm{mol}/L)=6.45\times (OD_{532}-OD_{600})-0.56\times OD_{450}$$

where *OD*_600_, *OD*_532,_ and *OD*_450_ denote the absorbance at wavelengths 600, 532, and 450 nm, respectively.

Chlorophyll content was determined by the extraction method [27], Beer-Lambert law, and the corresponding equations. During the measurement, the first step was to choose some mature leaves and remove all petioles. Then, they were cut into pieces, and 0.1 g leaves were put to a centrifuge tube (10 mL) filled with a mixed solution (ratio of the volume of acetone:absolute ethyl alcohol:deionized water = 4.5:4.5:1) to 10 mL which was put in dark for 48 h. These solutions were then diluted with the mixed solution to 25 mL, and the original mixed solution was set as the reference in the measurement. The specific absorption coefficients of the diluted solution under the illumination at wavelengths 663, 645, 440 nm were measured by a UV-2700 ultraviolet spectrophotometer from Shimadzu Corporation (Kyoto, Japan) and denoted as *OD*_663_, *OD*_645_, and *OD*_440_, respectively. Finally, the *OD*_663_, *OD*_645_, and *OD*_440_ were put into the following equations to work out the final results of contents of Chla, Chlb and carotenoid in mg/g, where V and W represent the volume of the solution (ml) and raw weight of the sample (g), respectively. The measurements of all chlorophyll content of the plant were repeated three times for getting average value.

(2) $$P_{\mathrm{Chla}}\left(\mathrm{mg}\right)=\frac{(12.7\times OD_{663}-2.69\times OD_{645})\times V}{\left(1000\times W\right)}$$

(3) $$P_{\mathrm{Chlb}}\left(\mathrm{mg}\right)=\frac{(22.9\times OD_{645}-4.68\times OD_{663})\times V}{\left(1000\times W\right)}$$

(4) $$P_{\mathrm{Chl}(a+b)}\;(\mathrm{mg})=\frac{(20.21\times OD_{645}+8.02\times OD_{663})\times V}{\left(1000\times W\right)}$$

(5) $$P_{\mathrm{Carotenoids}}\;(\mathrm{mg})=\frac{(4.7\times OD_{440}-0.27\times (20.21\times OD_{645}+8.02\times OD_{663}))\times V}{\left(1000\times W\right)}$$

### 2.5. Experiment Design

**Figure 3:**
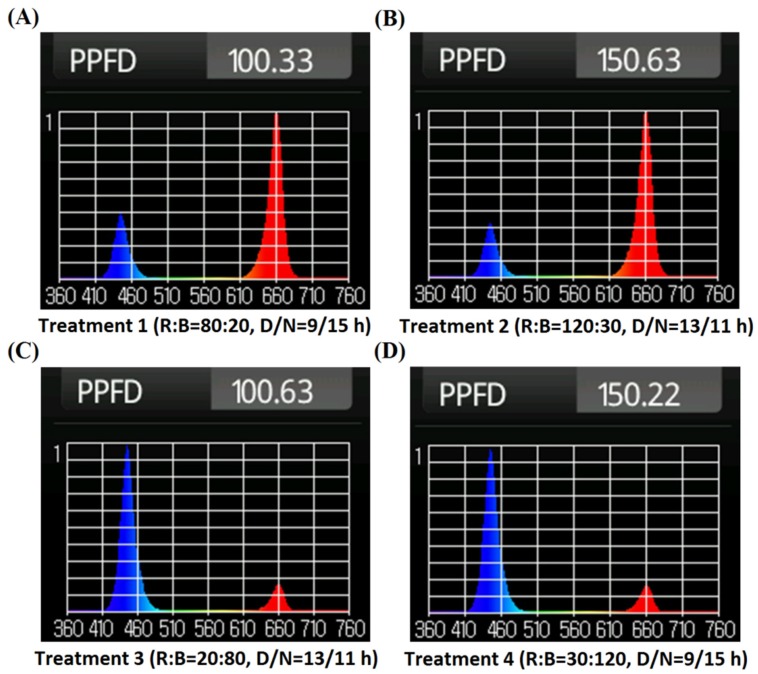
(**A**) The spectrum and total photosynthetic photon flux density (PPFD) of Treatment 1; (**B**) The spectrum and total PPFD of Treatment 2; (**C**) The spectrum and total PPFD of Treatment 3; (**D**) The spectrum and total PPFD of Treatment 4.

Under our experimental environment, in the preliminary experiments, when the light intensity was higher than 150 μmol∙m^−2^∙s^−1^, the spinach plant appeared burnt and died; when the lighting time exceeded 14 h per day, the plant showed early bolting. According to the preliminary experiments and the relevant researches before [16,17], the intensity of planting light for spinach growth should be set lower than 150 μmol∙m^−2^∙s^−1^, and the lighting time should not exceed 14 h/day. Thus, the experiment used the L_4_(2^3^) orthogonal table [28] regardless of interaction to analyze. As shown in Table 1, the ratios of red to blue light were set at 4:1 and 1:4, the light intensity levels were set at 100 and 150 μmol∙m^−2^∙s^−1^, and the photoperiods (day/night) were set at 9/15 and 13/11 h. The daily light integral (DLI) value and spectrum of each treatment group are shown in Table 1 and Figure 3. The spectra in Figure 1 were acquired by the hand-held spectrometer HR-350 made by HiPoint Corporation (Kaohsiung, China). The experiments with four treatment groups were executed three times repeatedly, and the average measured data were adopted for analysis.

### 2.6. Statistical Analysis

The data collected were subjected to analysis of variance (ANOVA) using MathCAD software. The analysis is based on least significant difference (LSD) [29] with a significance level at *p* ≤ 0.05. The results of the orthogonal test are analyzed following the regular procedure to get the weight vectors [18,19,20,21].

## 3. Results and Discussion

**Figure 4:**
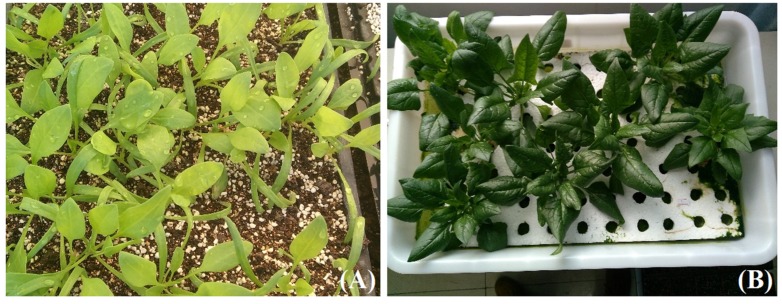
(**A**) Seedlings of spinach in tray; (**B**) spinach in plant growth chamber treated by light environment of Treatment 3, 31 days after transplant.

**Figure 5:**
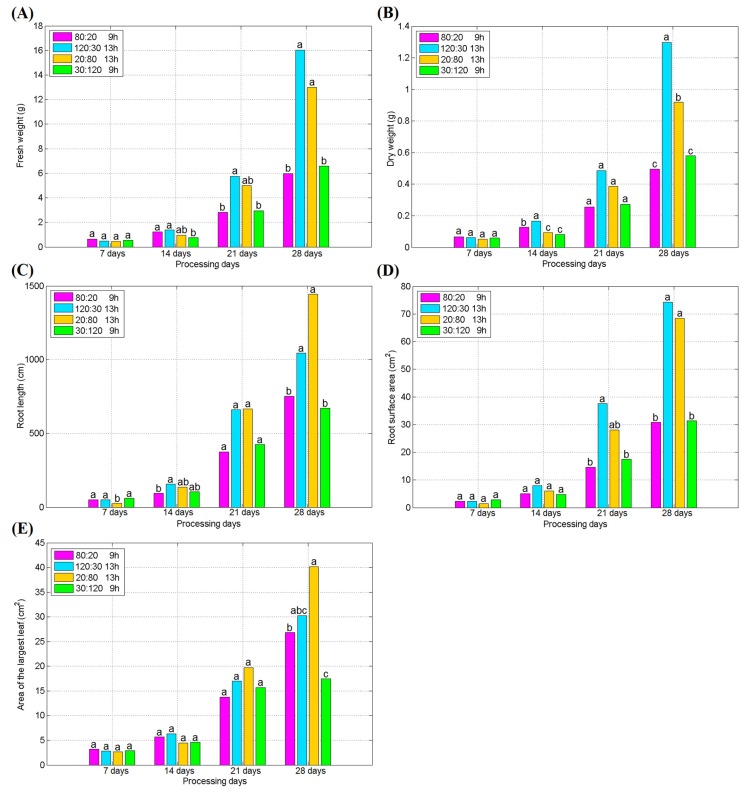
(**A**) The fresh weight of spinach treated with different light conditions after transplant; (**B**) the dry weight of spinach treated with different light conditions after transplant; (**C**) the root length of spinach treated with different light conditions after transplant; (**D**) the root surface area of spinach treated with different light conditions after transplant; (**E**) the area of the largest leaf of spinach treated with different light conditions after transplant. (Different letters represent a significant difference at α level of 0.05, *n* = 3).

Figure 4 shows two samples of spinach in our experiments. Figure 4A presents seedlings of spinach in trays, and Figure 4B shows the growing status of spinach plant by light treatment of Treatment 3, 31 days after transplant. Figure 5 shows the growth indexes of spinach treated with different light conditions after the transplant. The difference of each treatment group is not significant in the first seven days after transplant, except the root length of the plant in Treatment 3 (R:B = 1:4, PPFD = 100 μmol∙m^−2^∙s^−1^ and 13/11 h) showed lower than other test groups. On the 14th day after transplant, Treatment 2 (R:B = 4:1, PPFD = 150 μmol∙m^−2^∙s^−1^, and 13/11 h) had the highest fresh weight, dry weight, and root surface area among the test groups. Moreover, Treatment 1 with the same light ratio (R:B = 4:1) as Treatment 2 was also higher on the index of fresh weight and dry weight. On the 21st and 28th days after transplant, Treatments 2 (R:B = 4:1, PPFD = 150 μmol∙m^−2^∙s^−1^, and 13/11 h) and 3 (R:B = 1:4, PPFD = 100 μmol∙m^−2^∙s^−1^, and 13/11 h) showed the higher growth level than other two groups, and there was no significant difference between them on the most of the growth indexes, which implied that the photoperiod is a key factor for influencing the growth of spinach and the ratio of red and blue light is not so important. Therefore, when planting spinach in a plant factory, in order to increase yield, attention should be paid to the appropriate photoperiod.

Table 2 and Table 3 show the analysis of orthogonal test on growth indexes of spinach 28 d after transplant. The matrix analysis method and MathCAD software were applied for data processing.

Then, the weight vector of fresh weight can be worked out as Equation (6).

(6) $$X_{1}=\left[\begin{array}{ccc} 10.990 & 0 & 0\\ 9.797 & 0 & 0\\ \begin{array}{c} 0\\ 0\\ \begin{array}{c} 0\\ 0 \end{array} \end{array} & \begin{array}{c} \begin{array}{c} 9.489\\ 11.297 \end{array}\\ 0\\ 0 \end{array} & \begin{array}{c} \begin{array}{c} 0\\ 0 \end{array}\\ 6.271\\ 14.516 \end{array} \end{array}\right]\cdot \left[\begin{array}{ccc} \frac{1}{20.787} & 0 & 0\\ 0 & \frac{1}{20.787} & 0\\ 0 & 0 & \frac{1}{20.787} \end{array}\right]\cdot \left[\begin{array}{c} \frac{1.193}{11.246}\\ \frac{1.808}{11.246}\\ \frac{8.245}{11.246} \end{array}\right]=\left[\begin{array}{c} 0.056\\ \begin{array}{c} 0.050\\ 0.073\\ \begin{array}{c} 0.087\\ 0.221 \end{array} \end{array}\\ 0.512 \end{array}\right]$$

Next, the weight vectors of dry weight, root length, root surface area and area of the largest leaf are also calculated separately as follows: *X*_2_ = [0.085; 0.0771; 0.105; 0.139; 0.196; 0.404], *X*_3_ = [0.078; 0.0092; 0.144; 0.113; 0.208; 0.364], *X*_4_ = [0.029; 0.027; 0.033; 0.035; 0.266; 0.609], *X*_5_ = [0.008; 0.008; 0.250; 0.173; 0.213; 0.348]. Then, the average weight vector can be calculated as Equation (7).

*X =* (*X*_1_*+ X*_2_*+ X*_3_*+ X*_4_*+ X*_5_) /5 = [0.0512; 0.0496; 0.1210; 0.1094; 0.2208; 0.4474] (7)

According to the result, the weights for the two light quality levels are A1 = 0.0512 and A2 = 0.0496 and A1 is larger; the weights for the two light intensity levels are B1 = 0.1210 and B2 = 0.1094 and B1 is larger; the weights for photoperiod are C1 = 0.2208 and C2 = 0.4474 and C2 is larger. Thus, the optimal scheme for spinach growth in this experiment can be quickly determined as A1B1C2, that is, the light quality ratio is R:B = 4:1, the light intensity is 100 μmol∙m^−2^∙s^−1^, and the light period is 13/11 h. Thus, growth indexes for spinach are the best under the light parameter (R:B = 4:1, PPFD = 100 μmol∙m^−2^∙s^−1^, and 13/11 h) among all eight possible combinations in the range based on our experiment results, which is not one of the treatments listed in Table 1. That means that the orthogonal experiment is able to find an optimal combination outside of the designed treatments.

For the influence of the factors on the final results in general, the greater the weight of the factor is, the higher the degree of influence on the result. The total weights for light quality, intensity, and photoperiod are 0.1008, 0.2304, and 0.6682, respectively. Therefore, the influence on growth indexes of spinach according to the orthogonal test is C > B > A (photoperiod > light intensity > light quality). Thus, photoperiod has the greatest influence on the growth of spinach. However, it should not be too long (should be less than 14/10 h). The light intensity is the second important one, and the light quality has the weakest effect on the growth indexes of spinach.

**Figure 6:**
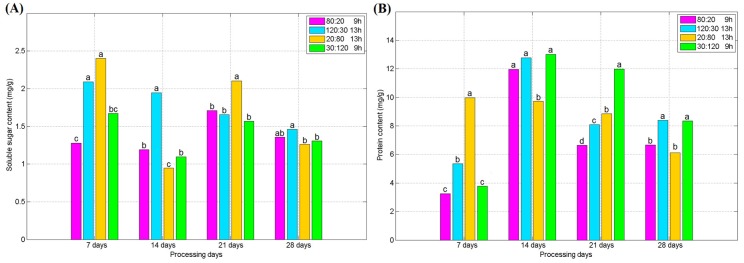
(**A**) Variation of soluble sugar content for different processing days; (**B**) variation of protein content for different processing days. (Different letters represent a significant difference at α level of 0.05, *n* = 3).

The variations of soluble sugar and protein contents in the experiments are shown in Figure 6. It can be seen that the content of soluble sugar in spinach rose first in the 0–7 d period and decreased in 7–14 d, then it rose again in 14–21 d, and decreased again in 21–28 d. During the 7–14 d period, due to the fast growth of the plant and the limited leaf area, the effect of respiration is higher than that of photosynthesis, the content of soluble sugar reduced. Furthermore, Treatment 2 accumulated the largest content of soluble sugar among these groups. According to the results from Treatments 2 and 4, photoperiod or light quality has an effect on the accumulation of soluble sugar in spinach. Figure 6B shows that Treatments 2 and 4 have accumulated more protein than the other two groups in the late stage of the cultivation. That may mainly depend on the higher light intensity in Treatments 2 and 4 compared to Treatments 1 and 3. Moreover, Treatment 2 has longer lighting time than Treatment 4, but there was no significant difference between them. Thus, the light intensity should be one of the important factors affecting the protein accumulation of spinach, but the effect of photoperiod is not as obvious.

As shown in Figure 5 and Figure 6, it can be seen that in the late growth stage of spinach, the leaves continued to grow rapidly, while the relative contents of soluble sugar and protein contents began to decrease, which is consistent with the development of plant leaves. Therefore, it is necessary to make a trade-off between the best yield and the best quality according to the purpose of the growers in the actual planting.

**Figure 7:**
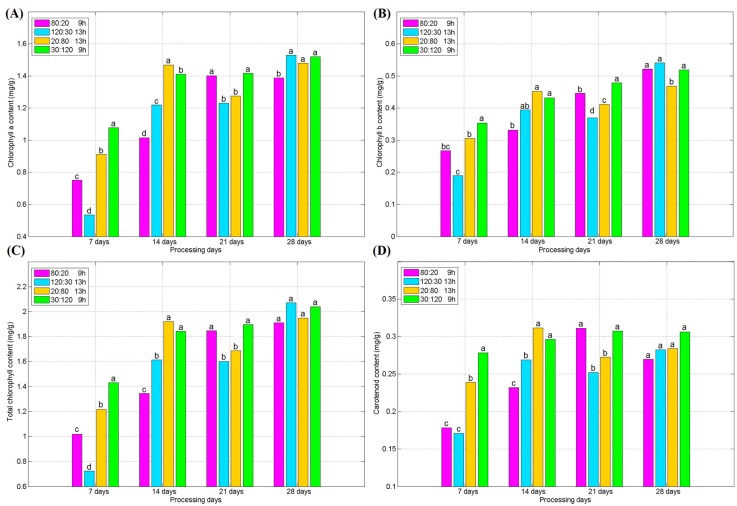
(**A**) Variation of Chlorophyll *a* for different processing days; (**B**) variation of Chlorophyll *b* for different processing days; (**C**) variation of total chlorophyll content for different processing days; (**D**) variation of Carotenoid for different processing days. (Different letters represent a significant difference at α level of 0.05, *n* = 3).

The changes of photosynthetic pigment levels in the experiments are shown in Figure 7. It can be seen that a higher blue light portion promotes the synthesis of chlorophylls *a*, *b* and carotenoids in the early stage of the spinach cultivation. Therefore, the content of chlorophyll and carotenoids in Treatments 3 and 4 is higher than that in Treatments 1 and 2 during the 0–14 d period. However, with the increase of growing time, this advantage gradually disappeared, and after 28 days, the difference of the photosynthetic pigments among different treatments tended to be insignificant.

**Figure 8:**
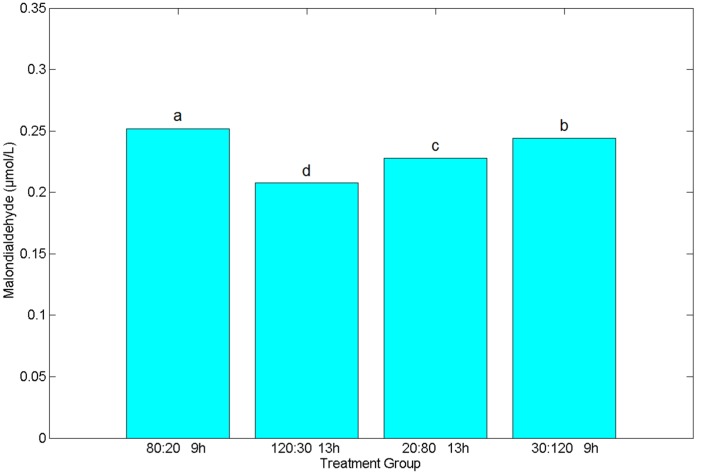
Malondialdehyde content of spinach after 28 days in different light environments. (Different letters represent a significant difference at α level of 0.05, *n* = 3).

Figure 8 shows the malondialdehyde content of each treatment after 28 days. The malondialdehyde content found in Treatment 1 was higher than in other treatments. There were also significant differences among the remaining treatments, with Treatment 2 having the lowest malondialdehyde content. As malondialdehyde content is the embodiment of the degree of membrane peroxidation in plant cells, the high content of malondialdehyde indicates that the membrane peroxidation of plant cells is high. In general, peroxidation occurs in plants under adverse conditions such as high temperatures, salinity, and high light intensity. This indicator suggests that Treatment 2 is the most suitable lighting environment for spinach growth among these four treatments, while Treatment 1 is the worst.

In order to comprehensively evaluate the quality indexes of spinach, a new scoring method is introduced in this paper to convert the indexes into a normalized dimensionless value for scoring. Assuming each sample value obeys the Gaussian distribution, the normalized *X* index can be calculated by Equation (8).

(8) $$z_{i}=\frac{x_{i}-\bar{x}}{\sigma /\sqrt{n}}$$

where $\bar{x}$ represents the sample mean, *σ* is the standard deviation, and *n* denotes the number of samples. The program is written using MathCAD, and its processing algorithm is shown in Algorithm 1.
**Algorithm 1.** The normalized scoring algorithm.**Input**: a vector of quality indexes of spinach *X***Output**: a matrix including the normalized weight *Z* and the normalized score *P**n*=cols(*X*); *ex*=mean(*X*); *σ*=Stdev(*X*); *σ^2^*=Var(*X*)for *i*∈*0*…*n*−1  $z_{i}=\frac{x_{i}-ex}{\sigma /\sqrt{n}}$
*c*=mean(*Z*); *s*=Stdev(*Z*); *s^2^*=Var(*Z*)$f(x)=\frac{1}{\sqrt{2\pi }s}e^{-\frac{{\left(x-c\right)}^{2}}{2s^{2}}}$ for *i*∈*0*…*n−1*  $p_{i}=\int_{-\infty }^{z_{i}}f(x)dx$ return (*Z*; *P*)

The normalized score of each index is obtained, and then added to obtain a total comprehensive score. Table 4 and Table 5 show the calculating results of each normalized score and the total comprehensive score for the spinach 28 days after transplant. Moreover, depending on the comprehensive ratings, Table 6 and Table 7 show the analysis of orthogonal tests on the quality indexes of spinach 28 d after transplant. Table 8 shows the analysis of variance for the orthogonal tests and determines that the errors did not influence the results of the analysis. According to the sum of comprehensive scores, the order of influence on quality indexes of spinach in the orthogonal test is B > C > A (light intensity > photoperiod > light quality), and the optimal light parameter for spinach quality in the experiment is A1B2C1 (R:B = 4:1, PPFD = 150 μmol∙m^−2^∙s^−1^, and 9/15 h) among all eight possible combinations in the range, which is again not one of the treatments listed in Table 1 and is different from that found in the growth index. That means that an optimal light mode for the growth index is not necessarily the optimal for the quality index. If farmers want to harvest spinach with a higher yield within a certain period of time, that is, spinach with larger leaf area and heavier weight, they need to pay more attention to the adjustment of the photoperiod and use a lower light intensity and a longer lighting time. If they do not mind the yield of vegetables, but want to improve the taste and nutrition of spinach products, they should pay more attention to the adjustment of light intensity and use a larger light intensity and a shorter lighting time. Therefore, the planting purpose determines what kind of lighting environment is selected in specific planting and whether it is quantity or quality priority.

## 4. Conclusions

In this research, the optimal parameters of artificial LED lighting for spinach were investigated through a self-designed plant growth shelf with uniform light. The orthogonal tests were applied to design the experiment with four groups to discover the effect of light intensity, quality, and photoperiod on the growth and quality indexes of spinach. According to the experimental results, for growth indexes including fresh weight, dry weight, root length and so on, photoperiod is the most influential factor, light intensity is the second, and the light quality is the least. The best light parameter (R:B = 4:1, PPFD = 100 μmol∙m^−2^∙s^−1^ and 13/11 h) among all eight possible combinations in the range was also determined. In order to evaluate the quality indexes, including the soluble sugar content, protein content and so on, a new scoring method was also introduced to make a comprehensive score. Then the parameter combination (R:B = 4:1, PPFD = 150 μmol∙m^−2^∙s^−1^ and 9/15 h) in the range was found as the optimal scheme for spinach quality within those parameters. However, there is a trade-off between the optimal parameters for growth and quality indexes. Thus, the growers of spinach are advised to get a balance between yield and quality of the plant. Lower light intensity and longer lighting time would lead to a higher yield that the farmers can have, and higher light intensity and shorter lighting time would improve the taste and nutrition of spinach products that the customer would prefer. Therefore, the planting purpose decides what kind of lighting environment is selected in specific planting and whether it has a quantity or quality priority.

## Figures and Tables

**Table 1:** The spectra and photoperiods of planting light for experiments.

	Red Light (μmol∙m^−2^∙s^−1^)	Blue Light (μmol∙m^−2^∙s^−1^)	Photoperiod (h)	Daily Light Integral (mol∙m^−2^∙d^−1^)
Treatment 1	80	20	9/15	3.24
Treatment 2	120	30	13/11	7.02
Treatment 3	20	80	13/11	4.68
Treatment 4	30	120	9/15	4.86

**Table 2:** Analysis of orthogonal test on growth indexes of spinach, 28 d after transplant.

	A (Light Quality)	B (Light Intensity)	C (Photoperiod)	Fresh Weight (g)	Dry Weight (g)	Root Length (cm)	Root Surface Area (cm^2^)	Area of the Largest Leaf (cm^2^)
Treatment 1	A1 (4:1)	B1 (100)	C1 (9/15 h)	5.963	0.495	750.749	30.803	26.845
Treatment 2	A1	B2 (150)	C2 (13/11 h)	16.016	1.298	1045.636	74.222	30.224
Treatment 3	A2 (1:4)	B1	C2	13.015	0.918	1445.394	68.472	40.121
Treatment 4	A2	B2	C1	6.578	0.580	670.054	31.382	16.135

**Table 3:** Analysis of orthogonal test on growth indexes of spinach, 28 d after transplant (continued).

	k1	k2	R	Excellent Level
Fresh weight (g)	k_1_^A^ = 10.990	k_2_^A^ = 9.797	1.193	A1
	k_1_^B^ = 9.489	k_2_^B^ = 11.297	1.808	B2
	k_1_^C^ = 6.271	k_2_^C^ = 14.516	8.245	C2
Dry weight (g)	k_1_^A^ = 0.897	k_2_^A^ = 0.749	0.148	A1
	k_1_^B^ = 0.707	k_2_^B^ = 0.939	0.232	B2
	k_1_^C^ = 0.538	k_2_^C^ = 1.108	0.57	C2
Root length (cm)	k_1_^A^ = 898.193	k_2_^A^ = 1057.724	159.531	A2
	k_1_^B^ = 1098.072	k_2_^B^ = 857.845	240.227	B1
	k_1_^C^ = 710.402	k_2_^C^ = 1245.515	535.113	C2
Root surfacearea (cm^2^)	k_1_^A^ = 52.513	k_2_^A^ = 49.927	2.586	A1
	k_1_^B^ = 49.638	k_2_^B^ = 52.802	3.164	B2
	k_1_^C^ = 31.093	k_2_^C^ = 71.347	40.254	C2
Area of the largest leaf (cm^2^)	k_1_^A^ = 28.535	k_2_^A^ = 28.128	0.407	A1
	k_1_^B^ = 33.483	k_2_^B^ = 23.180	10.303	B1
	k_1_^C^ = 21.490	k_2_^C^ = 35.173	13.683	C2

* Superscript letters A, B, C correspond to different factors in Table 2.

**Table 4:** Comprehensive score of quality indexes for spinach, 28 d after transplant.

	Soluble Sugar			Protein			Total Chlorophyll		
Treatment 1	0.505	0.515	0.627	0.369	0.140	0.269	0.164	0.112	0.117
Treatment 2	0.854	0.925	0.966	0.842	0.765	0.859	0.872	0.843	0.883
Treatment 3	0.089	0.158	0.207	0.121	0.128	0.117	0.264	0.193	0.317
Treatment 4	0.276	0.237	0.417	0.879	0.759	0.803	0.506	0.863	0.822

**Table 5:** Comprehensive score of quality indexes for spinach, 28 d after transplant (continued).

	Carotenoid			Malondialdehyde			Comprehensive Score		
Treatment 1	0.174	0.143	0.143	0.880	0.856	0.829	3.084	2.682	2.681
Treatment 2	0.559	0.312	0.383	0.068	0.124	0.061	3.625	3.272	3.616
Treatment 3	0.458	0.383	0.559	0.312	0.504	0.352	1.480	1.477	1.967
Treatment 4	0.746	0.994	0.785	0.654	0.713	0.815	3.435	3.870	3.937

**Table 6:** Analysis of orthogonal test on quality indexes of spinach 28 d after transplant.

	A (Light Quality)	B (Light Intensity)	C (Photoperiod)	Comprehensive Score			Sum
Treatment 1	A1 (4:1)	B1 (100)	C1 (9/15 h)	3.084	2.682	2.681	8.447
Treatment 2	A1	B2 (150)	C2 (13/11 h)	3.625	3.272	3.616	10.513
Treatment 3	A2 (1:4)	B1	C2	1.480	1.477	1.967	4.924
Treatment 4	A2	B2	C1	3.435	3.870	3.937	11.242

**Table 7:** Analysis of orthogonal test on quality indexes of spinach 28 d after transplant (continued).

K1	K2	k1	k2	R	Excellent Level
18.960	16.166	9.480	8.083	1.397	A1
13.371	21.755	6.686	10.878	4.192	B2
19.689	15.437	9.845	7.719	2.126	C1

* Importance: B > C > A; best combination: A1B2C1 (120:30 9/15 h).

**Table 8:** Analysis of variance for orthogonal test on quality indexes of spinach 28 d after transplant.

Variance Source	Sum of Squared Errors	Degree of Freedom	Mean Square	F value	Significance
A (Light quality)	0.651	1	0.651	10.50	≥ F_0.05_(1,8)^*^
B (light intensity)	5.858	1	5.858	94.48	≥ F_0.01_(1,8)^*^
C (photoperiod)	1.507	1	1.507	24.31	≥ F_0.01_(1,8)^*^
Errors	0.497	8	0.062		

* According to the F distribution threshold table F_0.05_ (1,8) = 5.32, F_0.01_(1,8) = 11.26.
